# Supplementary material for: Frequent detection of functional hyposplenism via assessment of pitted erythrocytes in patients with advanced liver cirrhosis
Source: PLoS One. 2022 Jul 18;17(7):e0271541. doi: 10.1371/journal.pone.0271541 (PMC9292104; doi:10.1371/journal.pone.0271541)
Supplement: Attachment — Submitted filename: Rebuttal letter.docx [file pone.0271541.s001.docx]

| Emily Chenette  Editor-in-Chief  PLOS ONE |  |  |
| --- | --- | --- |
|  | | Hamburg 23/04/2022 |

Dear Professor Chenette

Today we would like to resubmit our revised manuscript “**Frequent detection of functional hyposplenism via assessment of pitted erythrocytes in patients with advanced liver cirrhosis**” for peer review und possible publication as a Research Article in PLOS ONE.

We want to thank the reviewers for all of the many important suggestions. To address the issues raised by the reviewers, we revisited all patient files to collect and analyze additional follow-up data from the entire cohort. Furthermore, as suggested by reviewer 2, we took the opportunity to provide further evidence regarding a possible association between functional hyposplenism and portal hypertension in our cohort. Please find below a detailed point-by-point response to the reviewers` comments.

With regard to data availability: We chose to make the anonymized data available by reasonable request, as the German (and Hamburg city) data-protection authorities argue, that also anonymized patient-level data may lead to identification of certain patients. Therefore, we hesitate to make patient-level data available to the public because of legal worries.

We think that the resulting revised manuscript, which incorporates all of the reviewers´ suggestions, has significantly increased in quality and we hope that it is now acceptable in its current form for publication in PLOS ONE.

Thank you for your time and kind consideration.

Best regards,

Julian Schulze zur Wiesch	Malte Wehmeyer

**A point-by-point response to the reviewers´ comments:**

**Reviewer #1:** This is a good paper overall and the authors should be commended for their hard work. Few comments / suggestions:

- We thank reviewer #1 for their benevolent reception, critical review, and acknowledgment. Please find a detailed response to each suggestion below:

1. Figure 2 is not adding too much value or information - suggest removing it / adjusting the table.

- We agree with reviewer #1, therefore we removed (former) Figure 2 in the revised manuscript as the data are already presented in the results section and Table 1.

2. Further details on interference contrast microscopy should be added to the methods section - what microscope etc.

- We thank reviewer #1 for this suggestion. We added the respective information in the methods section.

3. You should add some pictures (?Appendix) of the pitted erythrocytes - the threshold of defining functional hyposplenism from this group is 15% and they have justified that - this is fine. Some other groups, including ours, have used a threshold of 4% and seeing so many patients with a pitted erythrocyte count >4% makes me wonder about over-counting some cells as pitted erythrocytes when they were not? [see BABY-HUG trial sickle cell, 10.1182/blood-2010-04-278747]. Hence, having pictures of pitted erythrocytes would help. Some of the referenes the authors used for arguing their threshold of 15% date back to 1976 - slightly outdated [reference 19].

- Thank you for the suggestion. We added an image of pitted erythrocytes (and HJB) as Figure 1 in the revised manuscript. Furthermore, we included the referenced paper in our introduction section. We now discuss a potential observer-based bias in the revised manuscript.

4. Agree with conclusions. The discussion is interesting. Do the authors have any other mechanistic reasons for hyposplenism in liver disease to comment on?

- Thank you. Previously, FH has been attributed to alcohol abuse in cirrhotic patients, which has already been discussed in the original manuscript. However, our data indicate that patients with other etiologies of cirrhosis also suffer from FH. Our results do not implicate another mechanistic reason for hyposplenism other than chronic portal hypertension. There might be additional functional immunological reasons (reduced clearance of microbial translocation etc.), however, we feel that such a discussion would be too hypothetical and not based on our data. Therefore, we would rather not include these valid discussion points in the discussion of the current manuscript.

**Reviewer #2:** The manuscript by Wehmeyer et al focuses on hyposplenism in advanced cirrhosis patients. They used measurement of pitted RBCs as a measure of splenic function. A few clarification or further details on some issues could be helpful, along with minor corrections as mentioned below:

- We would also tank reviewer #2 for their helpful and fair review f the current study.

Abstract
Use full forms of abbreviations at first use.

- We modified the abstract according to the reviewer´s suggestions. However, to improve the readability of the abstract, “functional hyposplenism” and “pitted erythrocytes” were spelled out throughout the abstract of the revised manuscript.

Structure the abstract into subheadings.

- We structured the manuscript with the subheadings “background”, “methods”, “results” and “conclusions”.

Association with portal hypertension not clear (from abstract).

- We agree with reviewer #2. The association of portal hypertension with liver stiffness measurements (by transient elastography) is now mentioned in the conclusion section of the revised abstract.

Introduction
Mention one of the studies on PE in detail, and as a function of FH.

- We thank the reviewer for this suggestion. We provide these details in the revised manuscript (Lammers et al. Am J Hematol 2012).

PE should be mentioned as one of the indirect markers of FH.

- We agree with reviewer #2. Therefore, we changed the sentence “Therefore, the occurrence of pitted erythrocytes is associated with poor splenic function”, to “…, pitted erythrocytes are regarded a reliable indirect marker of functional hyposplenism”.

Methods
Specify following:

Investigations done for etiologic work up of cirrhosis.

- We added the appropriate information in the revised manuscript.

Other causes of PE ruled out?

- Patients with a history of hematological co-morbidities and splenectomized patients were not included in the cirrhosis group. We added this clarification to the methods section of the revised manuscript. However, as portal vein thrombosis (PVT) is considered an alternative cause of functional hyposplenism and patients with liver cirrhosis often suffer from PVT, we further studied a possible association between FH and PVT: 4/28 patients with FH and 12/89 patients without FH had a PVT (P=1). We included this finding in table 1 of the revised manuscript.

Line 90- („pitted erythrocytes“) were… - correct the inverted comma.

- We corrected the inverted comma in the revised manuscript.

Line 95-‘percentage of erythrocytes with these abnormalities was calculated’- after counting _____ RBC.

- We added clarification of the calculation of the PE percentage.

Vaccination/anti-microbial policy in the patients.

- We evaluated the vaccination rates against pneumococci in our cohort. The corresponding paragraphs of the methods and results section of the manuscript were revised accordingly. Furthermore, the revised discussion section now clearly emphasizes the low vaccination rates. As this was an observational study, we did not change the vaccination schedule for patients in our study (in fact, this would be a clinical trial as per German jurisdiction).

It will be very helpful, if data is provided on past and study period infections in the study subjects- a better correlation of the overall outcome.

- We thank reviewer #2 for this important suggestion. Please see our response to the corresponding suggestion for the results section for further details.

Results:

Well described. Mention the following in results/tables:

Outcomes of the patients- with respect to infection rate observed in past and study period, infection related morbidity & mortality, other causes of mortality etc.

- We agree with reviewer #2. We collected the follow-up data by chart review of all patients. Interestingly, we were able to collect follow-up data from nearly all patients (112/117). However, due to the small sample size and due to study design (observational study without a pre-planned follow-up period or scheduled patient visits), we did not find a statistically significant association between FH and infection rates or other related variables. These limitations are now discussed transparently in the revised discussion section as well. Furthermore, the methods section was updated with regard to the follow-up data.

Correlation with portal hypertension.

- We re-organized the results section to further emphasize the evaluation of PE counts with portal hypertension. Transient elastography measurements were the only indicator of portal hypertension which was associated with the presence of functional hyposplenism, while the presence of ascites or esophageal varices was not associated with functional hyposplenism. Furthermore, we found an association between PE counts and the presence of portal hypertension according to the most recent Baveno VII criteria. In 13 patients hepatic venous pressure gradients were available from the time of study inclusion and patients with FH had higher HVPG. Due to the low number of patients (n=13), the results were statistically not significant. The discussion section was also updated according to these results.

Discussion:

Well written. Limitation of PE as a measure of splenic function.

- Thank you. The revised discussion section of the manuscript now clarifies, that PE is regarded as a marker of the phagocytic spleen function and that additional studies of immunization responses and B cell subsets are needed to evaluate the immunological function of the spleen.

Figures:
Mention the graph / statistical test depicted to show the results.

- We added the respective information to the figure legend.
